# Indices of embodied Neuroergonomic coupling: a theory and hypothesis framework for quantifying brain–body-environment dynamics in built space

**DOI:** 10.3389/fnhum.2026.1795326

**Published:** 2026-05-04

**Authors:** Joe Scanlin

**Affiliations:** Scanalytics, Madison, WI, United States

**Keywords:** built environment, dual-task gait, ecological validity, embodied cognition, mobile EEG, neuroergonomics

## Abstract

**Background:**

Built environments shape navigation, attention, and motor control through continuous brain–body-environment coupling, yet architecture, rehabilitation, and clinical mobility practice still lack a shared quantitative language for this interaction. Architects lack quantitative feedback linking spatial decisions to neural or gait outcomes; clinicians rely on episodic assessments that capture capacity snapshots but not continuous coupling. Portable multi-modal sensing, ecological neuroscience, and computational frameworks now make this problem newly tractable.

**Objective:**

To propose a theory-driven, testable framework of indices that operationalize embodied cognitive-motor coupling without reducing embodiment to a unitary resource-depletion model. The framework adopts an embodied-enactive stance and separates perceptual selection load, control and coordination load, and arousal regulation as partially overlapping mechanisms with distinguishable temporal, spectral, and recovery signatures.

**Framework:**

Six linked indices span four conceptual layers. Spatial Cognitive Demand (SCD) quantifies environment-level demand from pattern complexity, luminance variance, transition density, and visual-tactile alignment. Cognitive-Motor Fusion Index (CMFI) integrates neural demand, gait control cost, and instability into a bounded composite. Neuroergonomic Efficiency Quotient (NEQ) indexes motor performance relative to neural demand. Balance Recovery Coefficient (BRC) quantifies perturbation recovery quality. Gait-Cognition Coherence (GCC) measures frontal-theta to gait-phase coupling with context-dependent interpretation. Cognitive-Motor Headroom (CMH) estimates distance to an individualized operating boundary. Each index includes variable definitions, normalization constraints, and quality-governance requirements.

**Core hypotheses:**

Eight falsifiable hypotheses are advanced spanning five domains: environment-dependent shifts in coupled demand, mechanism-specific temporal dynamics, context-dependent interpretation of neural-gait coherence, conditional cross-domain transfer through domain-general control processes, and cross-level prediction from environmental structure to longitudinal mobility outcomes. These include predictions that higher-SCD environments elevate CMFI and reduce NEQ with stronger effects in lower-reserve groups, that perceptual-load and control-load manipulations yield separable spectral signatures, that elevated GCC reflects reduced automaticity in low-demand but adaptive recruitment in high-demand contexts, and that a multi-factor latent structure fits better than a single undifferentiated load factor.

**Conclusion:**

This manuscript does not report an inferential human-subject dataset. Its contribution is a conceptual and mathematical scaffold with a five-phase validation roadmap, reporting requirements for mobile EEG artifact governance, and a structured template for cumulative empirical testing across laboratories and populations.

## 1 Introduction

Everyday cognition is enacted in context: through continuous coupling among neural control, bodily action, and environmental affordances. In built environments, this coupling is constantly modulated by spatial layout, visual structure, material transitions, lighting variation, route predictability, and movement constraints. Yet design practice and clinical mobility workflows still rely mostly on qualitative judgment or late-stage outcomes, such as falls, rather than quantitative indicators of moment-to-moment cognitive-motor state.

This manuscript proposes a formal index framework for embodied neuroergonomic coupling in real-world navigation contexts. The objective is not to claim a finalized clinical instrument, but to define measurable constructs that can be empirically tested, falsified, and revised. Consistent with the Theory and Hypothesis article type, the paper separates conceptual definitions, provisional mathematical structure, falsifiable hypotheses, and validation requirements before applied deployment.

### 1.1 The measurement gap

There is still no routine equivalent of blood pressure, gait speed, or oxygen saturation for the coupled burden imposed by an environment on cognition and locomotion. Architects and designers make daily choices affecting cognitive-motor load, such as flooring patterns, luminance transitions, corridor geometry, signage density, and material changes, without a shared metric for evaluating how those choices alter neural control demand during movement. A space may be code-compliant and aesthetically successful yet still tax navigation, balance regulation, or compensatory control in ways that remain invisible until downstream failure.

Healthcare and rehabilitation practice face the same problem from another angle. Clinicians often work with episodic measures such as the Timed Up and Go, the Berg Balance Scale, or periodic cognitive screening (Podsiadlo and Richardson, 1991; Berg et al., 1992). These instruments are informative but provide only episodic snapshots. They do not directly describe how environmental context, locomotor control, and neural effort interact over time, nor do they fully resolve the gap between structured assessment and everyday mobility behavior (Hillel et al., 2019). For patients at risk of mobility decline, what matters is often not only whether gait is impaired in a clinic room, but how close the person operates to the edge of instability when exposed to ordinary route complexity, distraction, and perturbation (Li et al., 2018; Zukowski et al., 2020).

The measurement gap matters because the built environment is not merely a backdrop. It is part of the functional system through which navigation, balance, and adaptive behavior are realized (Wilson, 2002; Chemero, 2009; Coburn et al., 2020). If that coupling cannot be quantified, then design comparison, rehabilitation progression, and longitudinal monitoring all remain constrained by indirect proxies.

### 1.2 Why this problem is newly tractable

Three developments make the problem more tractable than in earlier eras. First, portable sensing has improved. Mobile EEG, instrumented walkways and floors, inertial sensing, synchronized video, and route-level environmental measurement now make it more feasible to study cognition during real movement than when most embodied-cognition debates were formulated (Debener et al., 2012; Gramann et al., 2011; Stangl et al., 2023). Second, dual-task gait and fall-risk research has matured to the point that cognitive involvement in locomotion is no longer a fringe claim but a documented feature of aging and clinical mobility (Al-Yahya et al., 2011; Li et al., 2018; Bishnoi and Hernandez, 2021; Wollesen et al., 2020). Third, architecture and environmental neuroscience have begun to demonstrate that interior form, visual complexity, and route features affect human behavior and brain dynamics, even though those effects are rarely translated into operational clinical or design metrics (Coburn et al., 2017; Banaei et al., 2017; Coburn et al., 2020).

### 1.3 Article-type commitments

The paper’s purpose is to define a coherent theory scaffold, specify the indices precisely enough to be tested, state where uncertainty remains, and make the validation burden explicit.

## 2 Theoretical foundation

### 2.1 Embodied-enactive commitment

The framework adopts an embodied-enactive stance: cognition is realized through ongoing perception-action loops constrained by body and environment, rather than solely by internal symbolic processing (Wilson, 2002; Barsalou, 2008; Chemero, 2009). Built space is therefore treated as part of the operative cognitive-motor system. The relevant question is not simply how much “load” a person carries in isolation, but how environmental affordances, bodily state, and neural control co-produce adaptive or maladaptive movement.

### 2.2 Operational role of attentional-control constructs

An embodied stance is compatible with attentional and control constructs, provided they are used carefully. Terms such as demand, allocation, and control load are used here as operational descriptors of regulatory requirements within a coupled system. They are not intended to imply a single depleting mental resource. The distinction matters because resource metaphors can create theoretical incoherence when treated as ontology rather than shorthand.

### 2.3 Mechanism alternatives and discriminative predictions

Several plausible mechanisms can contribute to embodied cognitive-motor coupling, and they should not be treated as interchangeable. The framework therefore separates at least three partially overlapping candidates:

1. *Perceptual selection load*: sensory competition under high scene complexity, clutter, or rapid feature transitions (Lavie et al., 2004; Lavie, 2005).
2. *Control and coordination load*: increased top-down regulation when locomotion must be coordinated with another task, obstacle negotiation, or strategic prioritization (Wickens, 2002; Wickens, 2008; Plummer and Eskes, 2015).
3. *Arousal and uncertainty regulation*: elevated vigilance under instability, unpredictability, or threat of error.

These mechanisms need not be mutually exclusive, but they make different predictions about decay time, transfer across tasks, neural signatures, and recovery dynamics (Lavie, 2005; Wickens, 2008; Cavanagh and Frank, 2014). Mechanism dissociation is therefore treated as a central empirical goal rather than an issue to be deferred until after index construction.

### 2.4 Cross-domain transfer and temporal persistence

Cross-domain transfer is not a given. Multiple-resource theory predicts that interference is strongest when tasks compete for similar modalities, codes, or processing stages (Wickens, 2002; Wickens, 2008). Accordingly, the present framework models cross-domain transfer as conditional rather than universal.

Instead, the framework proposes boundary conditions. Transfer should be strongest when navigation taxes domain-general executive operations such as updating, interference control, task-set coordination, or monitoring (Baddeley, 2000; Miyake et al., 2000; Cavanagh and Frank, 2014). It should be weaker, absent, or short-lived when the dominant burden is modality-specific and rapidly resolved once the route challenge ends. Effects on learning, knowledge work, and other downstream cognition are plausible extensions of the framework and remain active hypothesis-level targets for future empirical work.

The same logic applies to persistence. Perceptual selection effects may dissipate rapidly once environmental competition is removed, whereas arousal regulation or strategic over-control may linger longer in some individuals or contexts (Lavie, 2005; Wickens, 2008). Temporal persistence is therefore modeled as an empirical question, not as a settled property of the framework.

## 3 Conceptual architecture

### 3.1 Four linked layers

The proposed architecture has four linked layers:

1. *Environmental structure*: visual, material, luminance, transition, and route-predictability features.
2. *Neural control dynamics*: mobile EEG proxies of control engagement, selection, and motor regulation.
3. *Motor output stability*: gait timing, asymmetry, variability, center-of-pressure redistribution, and perturbation recovery.
4. *Adaptive capacity*: efficiency, coherence, and reserve or headroom relative to individualized operating limits.

The indices are modeled as partial observables of this chain, not as direct measurements of all latent mechanisms or all physiological subsystems. Vestibular, proprioceptive, subcortical, and cerebellar processes matter, but they are not directly measured by the current scaffold (Peterka, 2018; Stangl et al., 2023).

### 3.2 Properties of effective indices

An index is a compression function. It projects multidimensional, noisy, continuously varying data onto a scale that can still support interpretation and action. For the present framework, five properties matter:

1. *Legibility*: a reader should understand what higher or lower values mean.
2. *Sensitivity*: the index should move when the relevant coupled state changes.
3. *Specificity*: the index should not move indiscriminately in response to unrelated variation.
4. *Actionability*: different values should imply different follow-up questions or interventions.
5. *Trackability*: repeated measures should reveal trajectory rather than isolated snapshots.

These criteria are important because the aim is not simply to generate additional signals, but to build a shared language for describing how environments shape cognitive-motor state.

### 3.3 Conceptual architecture

**Figure 1:**
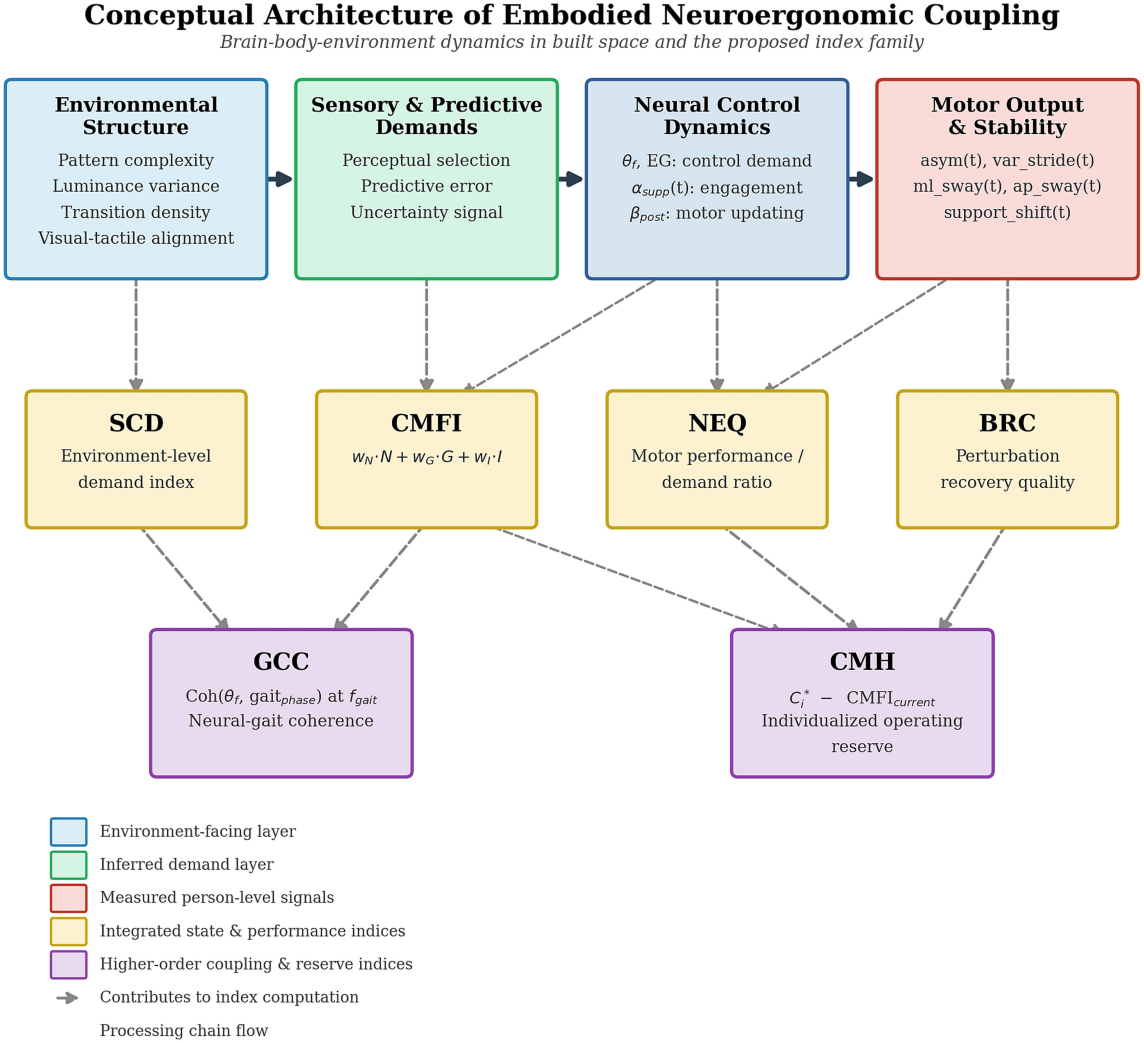
Conceptual processing chain targeted by the framework.

Figure 1 shows the conceptual processing chain targeted by the framework. Environmental structure gives rise to sensory and predictive demands, which are filtered through neural control dynamics and expressed in motor output and stability. The proposed indices do not all measure the same level: SCD is environment-facing; CMFI, GCC, and NEQ integrate across person-level signals; BRC captures perturbation response; and CMH references individualized operating reserve.

## 4 Index definitions and mathematical specification

### 4.1 Normalization and constraints

For a raw feature x , define:


$$\mathrm{norm}(x)=\text{clamp}(\frac{x-q_{10,\mathrm{ref}}}{q_{90,\mathrm{ref}}-q_{10,\mathrm{ref}}},0,1)$$ (1)

where $q_{10,\mathrm{ref}}$ and $q_{90,\mathrm{ref}}$ are reference percentiles for the relevant stratum, such as age, mobility status, task condition, or cohort. This formulation is intended to avoid ambiguous baseline language while still allowing both within-person and between-person interpretation.

For weighted composites, enforce:


$$w_{j}\geq 0\;\;\text{and}\;\;\sum_{j}w_{j}=1$$ (2)

unless otherwise stated. Fixed weights are not claimed as validated in this manuscript; they are to be estimated and stress-tested in later phases.

### 4.2 Core notation

Table 1 standardizes the main variables.

**Table 1:** Core variables and interpretation.

Symbol	Definition	Range
N(t)	Composite neural control-demand signal	[0, 1]
G(t)	Composite gait control-cost signal	[0, 1]
I(t)	Composite instability signal	[0, 1]
CMFI(t)	Coupled demand state index	[0, 1]
SCD	Environmental cognitive demand index	[0, 1]
M(t)	Composite motor performance signal	[0, 1]
NEQ(t)	Motor performance per unit neural demand	≥ 0
BRC	Recovery quality after perturbation	[0, 1]
GCC	Neural-gait coherence at gait frequency	[0, 1]
CMH	Distance to individualized operating boundary	real-valued

### 4.3 Neural demand signal

Define:


$$N(t)=w_{\theta }\cdot \theta_{f}(t)+w_{\alpha }\cdot \alpha_{\sup}(t)+w_{\beta }\cdot \beta_{\text{ctrl}}(t)$$ (3)

where:

1.
$\theta_{f}(t)$ is a normalized frontal-midline theta proxy for control demand, typically estimated from frontomedial channels or an equivalent source estimate (Cavanagh and Frank, 2014; Maidan et al., 2019).
2.
$\alpha_{\sup}(t)$ is a normalized parietal-occipital alpha suppression proxy for active selection and engagement (Klimesch, 2012).
3.
$\beta_{\text{ctrl}}(t)$ is a normalized sensorimotor beta modulation proxy for motor-set maintenance and updating (Engel and Fries, 2010).

The composite is not meant to imply that these signals collapse onto a single undifferentiated factor. Rather, it provides an interpretable, bounded summary of control demand that can later be tested against alternative model structures. One explicit alternative, to be evaluated in Phase 2, is that the components remain partially distinct and should enter downstream models separately or through latent-variable structure rather than as a simple observed composite.

### 4.4 Gait control and instability signals

Define a gait control-cost signal:


$$G(t)=0.5\cdot \text{asym}(t)+0.5\cdot {\mathrm{var}}_{\text{stride}}(t)$$ (4)

where asym(t) represents step asymmetry and ${\mathrm{var}}_{\text{stride}}(t)$ represents stride-time variability or a preregistered equivalent.

Define an instability signal:


$$I(t)=\frac{\begin{array}{l} \mathrm{norm}({\mathrm{ml}}_{\text{sway}}(t))+\mathrm{norm}({\mathrm{ap}}_{\text{sway}}(t))\\ +\mathrm{norm}({\text{support}}_{\text{shift}}(t)) \end{array}}{3}$$ (5)

where ${\mathrm{ml}}_{\text{sway}}$ , ${\mathrm{ap}}_{\text{sway}}$ , and ${\text{support}}_{\text{shift}}$ capture mediolateral sway, anterior–posterior sway, and rapid redistribution of support or pressure during stance and transition. These are proposed observables, not yet locked protocol outputs.

### 4.5 Cognitive-motor fusion index

Define:


$$\text{CMFI}(t)=w_{N}\cdot N(t)+w_{G}\cdot G(t)+w_{I}\cdot I(t)$$ (6)

Interpretation: an instantaneous coupled demand state, not a diagnosis and not a universal risk label.

The additive structure is intentionally conservative. It is more transparent than a multiplicative formulation, more robust to modality dropout, and easier to compare against regularized regression, SEM, or Bayesian hierarchical alternatives during validation, and serves as a provisional structural scaffold rather than a final empirically optimized estimator.

The following quality-handling conventions are recommended for any implementation:

1. Always report modality quality flags.
2. Always report a confidence channel alongside index values.
3. If one modality is unavailable, output a partial estimate explicitly rather than silently imputing a full score.

### 4.6 Spatial cognitive demand

Define:


$$\mathrm{SCD}=\lambda_{\mathrm{PC}}\cdot \mathrm{PC}+\lambda_{\mathrm{LV}}\cdot \mathrm{LV}+\lambda_{\mathrm{TD}}\cdot \mathrm{TD}+\lambda_{\mathrm{VTA}}\cdot (1-\mathrm{VTA})$$ (7)

where:

1. PC is pattern complexity.
2. LV is luminance variance.
3. TD is transition density.
4. VTA is visual-tactile alignment.

One central challenge for SCD is operational specificity Table 2 therefore makes the candidate raw observables explicit, even though final protocol lock is deferred to preregistration and empirical development.

**Table 2:** Candidate SCD operationalization schema.

Component	Candidate raw observable	Example units before normalization	Candidate extraction logic	Interpretation
PC	Contrast-weighted spatial-frequency complexity or spectral entropy from route imagery	entropy index; cycles/degree-weighted score	Compute route-level image descriptors from floor/wall regions in travel direction and aggregate over path segments	Higher values imply more perceptual competition
LV	Spatial and temporal variation in illumination	lux SD; coefficient of variation	Sample lux across route segments and transitions; quantify both absolute variance and abrupt change rate	Higher values imply more visual adaptation demand
TD	Material, texture, color, level, or edge transitions per route distance	counts per meter	Annotate route events from plans, video, or observational coding and normalize by path length	Higher values imply more frequent perceptual updating
VTA	Agreement between visual expectation and measured or rated surface properties	rubric score or agreement proportion	Compare visible surface cues to friction/compliance class, or to blinded assessor ratings of expected versus actual surface feel	Higher alignment lowers demand; misalignment increases prediction error

SCD will require demonstration of inter-rater reliability, inter-device reliability, and sensitivity to meaningful environmental differences before it can be considered validated. The present specification provides an operational scaffold rather than a settled standard.

### 4.7 Neuroergonomic efficiency quotient

Define:


$$\mathrm{NEQ}(t)=\frac{M(t)}{N(t)+\varepsilon }$$ (8)

where M(t) is a bounded motor performance composite and ε is a small stabilizing constant, for example 0.05.

Interpretation:

1. Higher NEQ means better motor output per unit neural demand.
2. NEQ is best interpreted within person, within stratum, or across matched contexts.
3. Apparent “good” motor behavior accompanied by very high neural demand should not be treated as efficient.

This index formalizes efficiency while making its main edge case explicit: denominator instability can create spurious inflation unless it is guarded analytically.

### 4.8 Balance recovery coefficient

For perturbation event k , define:


$${\mathrm{BRC}}_{k}=(1-E_{\mathrm{res},k})\cdot \exp\;(-\frac{t_{\mathrm{rec},k}}{\tau })$$ (9)

where:

1.
$t_{\mathrm{rec},k}$ is time to return below a preregistered instability criterion.
2.
$E_{\mathrm{res},k}$ is residual instability error in a fixed post-recovery window, normalized to [0, 1].
3.
τ is a recovery time constant defined before outcome analysis.

Aggregate:


$$\mathrm{BRC}=\text{median}({\mathrm{BRC}}_{k})\;\text{within standardized perturbation bins}$$ (10)

This formulation avoids rewarding larger perturbations irrespective of recovery quality and preserves a consistent direction of interpretation: higher BRC reflects better recovery quality.

### 4.9 Gait-cognition coherence

Define:


$$\mathrm{GCC}=\mathrm{Coh}(\theta_{f},{\text{gait}}_{\text{phase}})\;\mathrm{at}\;f_{\text{gait}}$$ (11)

Interpretation must be context-conditional:

1. In low-demand settings, elevated GCC with degraded NEQ may indicate compensatory over-control or reduced automaticity.
2. In high-demand settings, elevated GCC with preserved NEQ may indicate adaptive recruitment.

The meaning of elevated coherence is likely to be context-dependent rather than fixed. Accordingly, GCC should not be read univariately; it should be interpreted jointly with CMFI, NEQ, environmental demand, and performance context.

### 4.10 Cognitive-motor headroom

Define:


$$\mathrm{CMH}=C_{i}^{*}-{\text{CMFI}}_{\text{current}}$$ (12)

where $C_{i}^{*}$ is an individualized operating boundary estimated from challenge-response modeling or normative quantile models, not a universal fixed threshold.

Interpretation:

1. Positive CMH indicates operation below the modeled boundary.
2. Near-zero CMH indicates limited reserve.
3. Negative CMH indicates sustained operation beyond a modeled safe operating zone.

This formulation preserves the clinically and rehabilitation-relevant question of reserve without relying on universal thresholds.

## 5 Measurement architecture and implementation considerations

### 5.1 Multi-modal sensing stack

The framework is designed around synchronized measurement of environment, neural control, and locomotor output. A plausible validation stack includes:

1. Mobile EEG with sufficient channel density for artifact management and coarse source-informed interpretation.
2. Instrumented floor, walkway, pressure mat, or IMU-based gait sensing for timing, asymmetry, variability, and perturbation-related redistribution.
3. Environmental measurement tools such as calibrated imagery, lux measurement, route annotation, and surface-property characterization.
4. A synchronization strategy that supports sub-second alignment of neural, motor, and route-level events.

This outline is not intended to prescribe a single vendor system. It clarifies that the framework is inherently multi-level and that environmental demand cannot be inferred from EEG or gait alone.

### 5.2 Mobile EEG artifact and signal-quality governance

Mobile EEG during locomotion is artifact-prone, so artifact handling is treated here as a reporting-governance requirement rather than as a solved implementation detail (Gwin et al., 2010; Song and Nordin, 2021; Jacobsen et al., 2021; De Sanctis et al., 2020).

Minimum reporting expectations for future empirical papers should include:

1. *Acquisition controls*: electrode stabilization, cable minimization or wireless transmission, impedance targets, and motion-aware setup.
2. *Cleaning pipeline transparency*: ASR settings, ICA algorithm, component-classification method, and any gait-phase regression or template-removal steps (Chang et al., 2018; Chang et al., 2020; Delorme and Makeig, 2004; Pion-Tonachini et al., 2019).
3. *Objective ICA criteria*: number and proportion of removed components, ICLabel or equivalent class probabilities, residual variance or dipole fit when used, high-frequency EMG features, ocular signatures, and evidence of step-frequency locking.
4. *Signal-retention metrics*: proportion of usable epochs retained, exclusion rates, and pre/post-cleaning residual motion indices.
5. *SNR or contamination proxies*: for example, reduction in step-frequency contamination, channel-acceleration coupling, or out-of-band power inflation.
6. *Explicit uncertainty language*: no mobile EEG pipeline should be described as perfect artifact removal.

Dataset-specific SNR gains and component counts will emerge from future empirical work; what the present manuscript contributes is the reporting standard that such work should meet.

### 5.3 Task design, cohorts, and confound control

Several implementation considerations should guide future empirical studies:

1. Compare matched low and high-demand routes rather than relying only on abstract task labels.
2. Include both standardized-task and more naturalistic conditions to quantify ecological attenuation.
3. Track confounds such as time of day, medication status, fatigue, sleep quality, caffeine, pain, and prior route familiarity.
4. Include cohorts with differing reserve profiles, such as healthy younger adults, older adults, clinical mobility-impaired groups, or rehabilitation populations, depending on the target validation phase.

## 6 Falsifiable hypotheses

*H1*: Higher-SCD environments produce higher CMFI and lower NEQ for matched tasks, with stronger effects in lower-reserve groups.

*H2*: Perceptual-load and control-load manipulations produce separable temporal and spectral signatures in N(t) .

*H3*: Navigation-induced coupling effects show heterogeneous decay constants; individual decay slope predicts downstream cognitive-motor vulnerability.

*H4*: Elevated GCC predicts reduced automaticity only in low-SCD contexts with concurrent NEQ reduction; in high-SCD contexts, elevated GCC can reflect adaptive recruitment.

*H5*: Lower BRC predicts prospective near-fall and fall risk beyond steady-state CMFI alone.

*H6*: SCD explains between-environment variance in CMFI and NEQ after adjustment for person-level baseline capacity.

*H7*: Cross-domain interference is conditional rather than universal: strongest transfer occurs when navigation challenges recruit domain-general executive coordination rather than only modality-specific visuospatial processing.

*H8*: A latent structure with separable but correlated factors for environmental demand, neural control demand, and motor stability fits better than a single undifferentiated load factor.

## 7 Validation roadmap

### 7.1 Phase 0: specification lock and preregistration

1. Freeze equations, preprocessing steps, exclusion criteria, and outcome definitions.
2. Lock candidate operationalizations for PC, LV, TD, and VTA.
3. Pre-register model-comparison rules, alternative composite structures, and missing-data handling.

### 7.2 Phase 1: construct validity and reliability

1. Test–retest reliability across sessions.
2. Inter-rater and inter-device reliability for SCD components.
3. Convergent and divergent validity against established gait, balance, and cognitive measures.
4. Manipulation checks showing that controlled route changes alter the intended components.

### 7.3 Phase 2: parameter estimation and construct structure

1. Estimate weights using regularized regression, Bayesian hierarchical models, or equivalent transparent methods.
2. Compare observed-composite and latent-variable formulations.
3. Evaluate multicollinearity and apply shrinkage or orthogonalization where required.
4. Report parameter uncertainty rather than only point estimates.

### 7.4 Phase 3: predictive validity

1. Test prospective outcomes such as near-falls, falls, mobility decline, or rehabilitation response.
2. Report discrimination, calibration, and decision-utility metrics.
3. Evaluate whether recovery metrics add information beyond steady-state metrics.

### 7.5 Phase 4: ecological validity and transportability

1. Compare laboratory-standardized and field-deployed performance.
2. Quantify domain shift from standardized tasks to real facilities and real routes.
3. Recalibrate transparently when transporting models across populations, sites, or sensors.
4. Test whether route-level indices remain interpretable under real-world distraction, crowding, and variable environmental noise (Zukowski et al., 2020; Zukowski et al., 2021; Stangl et al., 2023).

## 8 Scope, limits, and boundary conditions

### 8.1 Boundary conditions and exclusions

Several boundaries should be stated explicitly. The framework does not claim:

1. That the proposed indices are clinically validated.
2. That a universal CMFI threshold exists.
3. That scalp EEG directly captures all relevant subcortical or vestibular processes.
4. That cross-domain effects on learning, productivity, or creativity have already been demonstrated.
5. That any specific sensor vendor or proprietary platform is necessary for implementation.

### 8.2 Population and context dependence

Application to new cohorts, such as stroke, Parkinson’s disease, dementia, or specialized occupational groups, requires external validation and likely recalibration. The same environment can challenge different people in different ways depending on reserve, strategy, anxiety, motor impairment, and route familiarity (Li et al., 2018; Mirelman et al., 2019).

### 8.3 Ecological validity

Laboratory tasks and controlled walking corridors differ substantially from real-world navigation. Real routes contain competing pedestrians, unexpected events, acoustic distraction, wayfinding decisions, and self-selected strategy shifts. A practical advantage is that many target built environments, such as healthcare corridors, rehabilitation facilities, and public transit concourses, are physically stable and reproducible, making systematic field validation feasible. The framework therefore includes an ecological-validity phase rather than treating field deployment as a trivial extension of lab work (Holleman et al., 2020; Hillel et al., 2019; Stangl et al., 2023). Transportability must be demonstrated, not presumed.

### 8.4 Translational implications

If validated, the most defensible translational uses are:

1. Comparing environmental variants in rehabilitation and healthcare settings,
2. Tracking individualized rehabilitation progression and reserve,
3. Longitudinal monitoring of cognitive-motor resilience,
4. Generating testable hypotheses about how route complexity contributes to mobility vulnerability.

Broader claims about workplaces, schools, or commercial environments should remain bounded and hypothesis-level until they are tested with appropriate outcome measures.

## 9 Discussion

The framework is intended to give brain–body-environment coupling a more explicit quantitative vocabulary without collapsing distinct mechanisms into a single generic load construct. Its value will depend on whether future studies show that the proposed indices are discriminative, interpretable, and transportable across contexts.

Several features are meant to support that goal. Environmental demand, neural control, motor stability, recovery dynamics, and reserve are separated conceptually rather than forced into a single score. The equations (Equations 1–12) are explicit, alternative structures remain open to empirical comparison, and the validation burden is specified in advance. These choices are intended to facilitate comparison, falsification, and revision rather than premature closure.

The broader motivating argument is that built environments can impose meaningful demands on navigation, balance regulation, and control recruitment, while existing clinical or design vocabularies remain poorly equipped to describe those demands in real time (Banaei et al., 2017; Li et al., 2018; Zukowski et al., 2020). A coherent index language could therefore support rehabilitation, mobility monitoring, and evidence-guided environmental comparison, provided that validation is carried out transparently and conservatively (Hillel et al., 2019; Stangl et al., 2023).

Spatial neuroergonomics sits at the interface of mobility science, environmental design, embodied cognition, and mobile neuroimaging (Gramann et al., 2011; Coburn et al., 2017; Li et al., 2018; Stangl et al., 2023). That interface is analytically useful because these fields still rely on partially incompatible vocabularies for closely related phenomena.

The practical urgency of this work is underscored by demographic trends. The population aged 65 and older is growing rapidly in the United States and globally, with projections indicating that older adults will constitute an increasingly large share of the population navigating complex built environments over the coming decades. Age-related declines in gait stability, executive function, and multisensory integration mean that a growing proportion of the population will navigate built environments with diminished cognitive-motor reserve (Li et al., 2018; Mirelman et al., 2019). Falls already constitute a leading cause of injury-related morbidity and mortality among older adults, with dual-task gait measures increasingly recognized as prospective indicators of fall risk (Lundin-Olsson et al., 1997; Gillain et al., 2019). The healthcare costs associated with fall-related injuries are expected to rise in parallel with population aging. A quantitative framework that can detect when and where built environments tax cognitive-motor capacity beyond safe operating limits could inform evidence-based design standards for healthcare facilities, assisted-living environments, public transit systems, and age-friendly communities. More broadly, the ability to characterize person-environment coupling in real time opens pathways for early identification of mobility decline, individualized rehabilitation programming, and proactive environmental modification before adverse events occur.

## 10 Conclusion

This manuscript presents a theory-driven, mathematically explicit framework for embodied neuroergonomic coupling in built environments. Its contribution is not a validated clinical instrument, but a falsifiable conceptual architecture intended to support transparent empirical testing, independent replication, and iterative refinement.

The value of the framework will depend on whether future studies show that its indices capture something real, discriminative, and useful about how environments, bodies, and neural control interact during movement. Where empirical results diverge from predictions, the staged validation roadmap provides a structured path for transparent revision and iterative improvement.

## Data Availability

The original contributions presented in the study are included in the article/supplementary material, further inquiries can be directed to the corresponding author.
